# Mining the transcriptome for rare disease therapies: a comparison of the efficiencies of two data mining approaches and a targeted cell-based drug screen

**DOI:** 10.1038/s41525-017-0018-3

**Published:** 2017-04-24

**Authors:** A. J. Mears, S. C. Schock, J. Hadwen, S. Putos, D. Dyment, K. M. Boycott, Alex MacKenzie

**Affiliations:** 1 Children’s Hospital of Eastern Ontario (CHEO) Research Institute, University of Ottawa, Ottawa, ON K1H 8L1 Canada; 2 Department of Cellular and Molecular Medicine, University of Ottawa, Ottawa, ON Canada; 3 Department of Genetics, Children’s Hospital of Eastern Ontario, Ottawa, ON Canada

## Abstract

Most monogenic diseases can be viewed as conditions caused by dysregulated protein activity; therefore, drugs can be used to modulate gene expression, and thus protein level, possibly conferring clinical benefit. When considering repurposing drugs for loss of function diseases, there are three classes of genetic disease amenable to an increase of function; haploinsufficient dominant diseases, those secondary to hypomorphic recessive alleles, and conditions with rescuing paralogs. This therapeutic model then brings the questions: how frequently do such clinically useful drug–gene interactions occur and what is the most rapid and efficient route by which to identify them. Here we compare three approaches: (1) mining of pre-existing system-wide transcriptomal datasets such as Connectivity Map; (2) utilization of a proprietary causal reasoning engine knowledge base; and, (3) a targeted drug screen using clinically accepted agents tested against normal human fibroblasts. We have determined the validation rate of these approaches for 76 diseases (i.e., in vitro fibroblast mRNA increase); for the Connectivity Map, approximately 5% of tested putative drug–gene interactions validated, for causal reasoning engine knowledge base the rate was 10%, and for the targeted drug screen 9%. The degree of overlap between these methodologies was low suggesting they are complementary not redundant approaches to identify putative drug-gene interactions. Although the validation rate was low, a number of drug–gene interactions were successfully identified and are now being investigated for protein induction and in vivo effect. This analysis establishes potentially valuable therapeutic leads as well as useful benchmarks for the thousands of currently untreatable rare genetic conditions.

## Rare diseases: drug re-discovery

Canadian researchers use three different approaches to identify drugs that could be repurposed to treat rare genetic diseases. Prompted by the growing gap between known rare diseases and therapies and postulating that diseases might be treated with drugs that modulate levels of disease-related proteins, Alex Mackenzie at the University of Ottawa and colleagues, studied the impact of 300 clinically approved drugs on 75 rare disease associated genes. Analyses of Connectivity Map gene expression data, published texts and a cell-based screening assay revealed thousands of putative and potentially clinically beneficial interactions. Although just 5–10% altered protein expression, the different approaches were complementary rather than redundant methodologies identifying potentially useful starting points on the path to rare disease therapy.

## Introduction

The estimated 7000 monogenic diseases although individually rare are major contributors to human morbidity and mortality collectively affecting approximately 2% of the global population.^1^ For example, rare diseases account for nearly twice the aggregate number of years that lives are shortened by diabetes and almost four times those due to infections.^2^ Rare genetic diseases thus represent a dramatic unmet diagnostic and therapeutic need. Roughly half of the 7000 rare monogenic diseases have been genetically characterized.^1, 3^ The remaining diseases are being solved largely due to the work of national and international consortia such as Care4Rare Canada (http://care4rare.ca/) and the Centers for Mendelian Genomics (3) with collaborative platforms such as the Matchmaker Exchange (http://www.matchmakerexchange.org/.^4^ However, the development of rare disease treatment lags far behind the rate of rare disease diagnosis; approximately 500 therapies have been approved for rare diseases (Europe and USA combined^5^). Moreover, the rate of drug development for rare diseases is slow, due to factors such as extreme disease rarity and obscure disease pathogenesis.

One alternative to the costly and time-intensive drug discovery process is to repurpose clinically approved compounds for treatment of rare diseases.^6^ Often, rare monogenic diseases can be viewed as dosage problems caused by supra-physiologic or infra-physiologic levels of functional gene-product, usually altering protein activity. Under this model, moderating the dosage problem by modulating mRNA and thus protein levels is a potential strategy to repurpose drugs for rare monogenic diseases. This may involve the upregulation of mutated recessive disease genes encoding proteins with residual enzymatic activity (so called hypomorphic alleles), of genes that functionally recapitulate mutated recessive disease genes (rescuing paralog^7^) or of genes that cause disease when haploinsufficient. Conversely, pathogenically increased gene dosage (e.g., gain of function dominant mutations, gene duplication) may be countered by gene downregulation.

The gene-dosage therapeutic model relies on the identification of therapeutically relevant drug-gene interactions in which a clinically approved drug modulates mRNA and protein levels. Several approaches to identify such drug-gene interactions exist, yet assessment of and comparison between these techniques have not been conducted. Extensive pharmacologic transcriptome datasets (nearly 4000 representing approximately 2 million samples) are accessible through the Gene Expression Omnibus (GEO) database (http://www.ncbi.nlm.nih.gov/geo/ ^8^). One of the largest sets of data is provided by Connectivity Map (CMAP) which generated microarray transcriptome profiles for 3 different cancer cell lines (leukemia (HL60), breast (MCF7), and prostate (PC3)) treated with over 1200 drugs.^9, 10^ Another bioinformatic-based approach to identify putative drug-gene interactions is literature-mining; Pfizer has developed a proprietary advanced algorithm for text-mining called the Causal Reasoning Engine (CRE),^11, 12^ which operates against a knowledge base integrating causal interactions from several sources. We have employed the latter two approaches as well as an in-house drug screen on normal human fibroblasts using a curated publicly available library of FDA approved compounds to establish the frequency with which such drug-gene interactions can be first identified and then validated (modulation of mRNA in cell culture), as well as the degree of overlap between these three approaches.

## Results

### Identifying potential therapeutic targets

We set out to identify a subset of rare diseases that were potentially amenable to mRNA (and by extension protein) modulation. We reviewed the rare disease databases OMIM ^3^ and Orphanet ^13^ to identify diseases with a potential mRNA target: (1) autosomal dominant rare diseases caused by haploinsufficiency; (2) recessive diseases where there is evidence that hypomorphic alleles encode proteins with residual function that localize normally in the cell; and (3) diseases where there exists another gene that may functionally recapitulate the disease gene’s function (rescuing paralog). Next, an expert clinical group (KMB, DD, and clinical members of the FORGE Canada Consortium, ^14^) further reduced this list by looking for: (1) the existence of a pre-symptomatic period and/or the possibility of reversibility; (2) degree of unmet medical need; and (3) known Canadian patients affected with the disorder to arrive at a list of 76 diseases for study (Table 1).

**Table 1:** The 76 diseases and associated 75 genes analyzed in this study (classified as haploinsufficient; paralog rescue, or hypomorph categories)

Gene	Disorder	OMIM#	Fibroblast screen?	CMAP data?	CNS disease?
*HAPLOINSUFFICIENT*					
AFG3L2	Spinocerebellar ataxia type 28	610246	yes	yes	yes
ATP1A2	Familial hemiplegic migraine type 2	602481	no	yes	yes
COL6A1	Bethlem myopathy	158810	yes	yes	no
CSF1R	Hereditary diffuse leukoencephalopathy with spheroids	221820	no	yes	yes
GRN	Frontotemporal lobar degeneration with ubiquitin-positive inclusions	607485	no	yes	yes
ITPR1	Spinocerebellar ataxia type 15	606658	yes	yes	yes
MAPT	Dementia, frontotemporal, with or without parkinsonism	600274	no	yes	yes
MPZ	Charcot-Marie-Tooth disease type 1B	118200	no	yes	yes
NKX2-1	Chorea, hereditary benign	118700	no	yes	yes
OPA1	Optic atrophy type 1	605290	yes	yes	yes
PMP22	Hereditary neuropathy with liability to pressure palsies	162500	yes	yes	yes
SCN1A	Dravet syndrome	607208	yes	yes	yes
SLC2A1	GLUT1 deficiency	612126	yes	yes	yes
SMAD3	Familial thoracic aneurysm/ Loeys Dietz syndrome type 3	613795	yes	yes	no
SPAST	Hereditary spastic paraparesis type 4	182601	yes	yes	yes
*PARALOG RESCUE (* *paralog target* *)*					
ABCD1 (ABCD2)	X-adrenoleukodystrophy	300100	no	yes	yes
DDHD2 (DDHD1)	Complex hereditary spastic paraplegia (SPG 54)	615033	yes	no	yes
FBN1 (FBN2)	Marfan syndrome	154700	yes	yes	no
LIMS2 (LIMS1)	Limb girdle muscular dystrophy with cardiomyopathy type, 2W	616827	yes	yes	no
SLC39A8 (SLC39A14)	Congenital disorder of glycosylation, type IIn	616721	yes	yes	yes
*HYPOMORPH*					
ACADVL	ACADVL deficiency (VLCAD)	201475	no	yes	no
AGPAT2	Lipodystrophy, congenital generalized, type 1	608594	no	yes	no
AGXT	Primary hyperoxaluria	259900	no	yes	no
ALDH18A1	Cutis laxa, autosomal recessive, type IIIA	219150	no	yes	yes
AMACR	Apha-methylacyl-CoA racemase deficinecy	614307	no	yes	yes
ARSA	Metachromatic leukodystrophy	250100	yes	yes	yes
ASAH1	Farber disease	228000	yes	yes	yes
	Spinal muscular atrophy with progressive myoclonic epilepsy	159950			
ASL	Argininosuccinic aciduria	207900	no	yes	yes
ASPA	Canavan disease	271900	no	yes	yes
ATP7A	Occipital horn syndrome	304150	yes	yes	no
ATP7B	Wilson disease	277900	yes	yes	yes
BCKDHA	Maple syrup urine disease (intermediate), type Ia	248600	yes	yes	yes
BCKDHB	Maple Syrup urine disease (Intermediate), type Ib	248600	yes	yes	yes
BSCL2	Lipodystrophy, congenital generalized, type 2	269700	yes	yes	no
CLN3	Ceroid lipofuscinosis type 3 (Batten disease)	204200	yes	yes	yes
CPT2	Carnitine palmitoyltransferase II deficiency	255110	yes	yes	no
CTSA	Galactosialidosis	256540	yes	yes	yes
DDHD2	Complex hereditary spastic paraplegia (SPG 54)	615033	no	yes	yes
EIF2B5	Central hypomyelination and vanishing white matter disease	603896	yes	yes	yes
ETFA	Glutaric acidemia type 2	231680	yes	no	no
FH	Fumarase deficiency	606812	yes	yes	yes
FKRP	Limb girdle muscular dystrophy type 5C	607155	no	yes	no
GAA	Glycogen storage disease type 2/Pompe	232300	yes	yes	no
GALC	Krabbe disease	245200	yes	yes	yes
GALNS	Mucopolysaccharidosis IVA	612222	yes	yes	no
GALT	Galactosemia	230400	yes	yes	no
GBE1	Glycogen storage disease type IV	232500	yes	yes	no
GLB1	Mucopolysaccharidosis type IVB (Morquio)	253010	yes	yes	no
	GM1-gangliosidoses Type 3	230650			
GUSB	Mucopolysaccharidoses VII	253220	yes	yes	yes
HARS	Usher syndrome	614504	yes	yes	yes
HEXA	Tay Sachs disease	272800	yes	yes	yes
HEXB	Sandhoff disease	268800	yes	yes	yes
HPRT1	Kelley-Seegmiller syndrome	300323	yes	yes	no
HSD11B2	Mineralocorticoid Excess	218030	no	yes	no
HSD17B4	D-bifunctional protein deficiency	261515	yes	yes	yes
IDS	Mucopolysaccharidoses II	309900	yes	yes	yes
IDUA	Mucopolysaccharidoses I	607014	no	yes	yes
MAN2B1	Alpha-mannosidosis type I	248500	yes	yes	yes
MUT	Methylmalonic aciduria, mut type	251000	yes	yes	yes
NEU1	Sialidosis type 1	256550	yes	yes	yes
OTC	Ornithine transcarbamylase deficiency	300461	no	yes	yes
PHYH	Adult Refsum disease	266500	yes	yes	yes
PLP1	PLP1-related disorders	312080	no	yes	yes
PMM2	Congenital disorder of glycosylation type 1C	212065	yes	yes	yes
POLR3A	Leukodystrophy, hypomyelinating, 7	607694	yes	no	yes
PPT1	Ceroid lipofuscinosis type1	600722	yes	yes	yes
SACS	ARSACS	270550	yes	yes	yes
SBDS	Shwachman-Bodian-Diamond syndrome	260400	yes	no	no
SCARB2	Action myoclonus renal failure syndrome	254900	yes	yes	yes
SGSH	Mucopolysaccharidoses III	252900	no	yes	yes
SLC16A2	Allan-Herndon-Dudley syndrome	300523	no	yes	yes
SLC52A2	Brown-Vialetto-VanLaere Sensory neuropathy	614707	no	yes	yes
SLC6A8	Creatine transporter deficiency	300352	no	yes	yes
SUMF1	Multiple sulfatase deficiency	272200	yes	no	yes
TYMP	Mitochondrial DNA depletion syndrome type 1	603041	yes	yes	yes
*METABOLIC BYPASS*					
NEU1	Tay Sachs disease	272800	yes	yes	yes

Of the 76 diseases (associated with 75 different genes), 57 can be caused by hypomorphic mutations (in 55 genes) that might be improved by over-expressing the mutated partially functional protein. We believe that the upregulation of mRNA/protein is a credible treatment modality in such cases given that a modest increase of enzyme activity in a recessive disease may have a profound impact clinically (28). Care was taken to avoid diseases in which the majority of associated mutations resulted in mislocalization or 0% activity. Fifteen of the diseases are haploinsufficient autosomal dominantly inherited conditions caused by a mutation in a single allele; these diseases likely represent the most promising class for pharmacologic gene induction with the remaining non-mutated allele serving as the drug target; we are aware of at least two examples employing this approach in the literature.^15, 16^ In this instance, care has been taken to identify conditions where there is good evidence that the causal mutation is null and does not encode a protein with either dominant negative or gain of function properties. In 5 of the 76 diseases, our goal was the over-expression of a paralog; the concept of treating a genetic disease by modulating expression of a gene that functionally compensates (at least partly) the mutation gene is a well-known but comparatively unused therapeutic approach exemplified by SMN2 induction in spinal muscular atrophy (7). In the final instance of Tay Sachs disease, the neuraminidase genes sit on a metabolic bypass pathway which may moderate the clinical severity (29) although recent work has called this interpretation into question (30).

### Mining of CMAP for drug conferred gene induction

Broad differences of mRNA “responsiveness” to the drugs were observed when mining the CMAP data; in general drugs such as histone deacetylase inhibitors (HDACi’s) induced the most genes while others had little impact on the transcriptome (Supplementary Table 1). The response of 70 of the 75 target genes (Table 1) to the 149 CMAP drugs also found in the 310 compounds that were later tested in our fibroblast screen was extracted from the CMAP dataset; only two genes failed to be increased by at least one drug. Of the 10,430 possible drug-gene interactions (70 genes × 149 drugs) recorded in CMAP, 970 demonstrated induction (9.3%) in at least one of the three cancer cell lines tested. It should be noted the significant majority of these hits were singletons (89%) and a much smaller fraction displayed induction in two (10%) or all three cell lines (1%; see Table 2; Supplementary Table 2; columns LC-LK).

**Table 2:** Assessment in the fibroblast screen, presence in Connectivity map database and CNS involvement

GENE	DRUG	Validation response	CMAP	CREBK	FScreen
ATP1A2	Calcitriol	[++]		z	n/a
	Biperiden	++	x		n/a
ITPR1	Dexamethasone	++	x	z	
	Dasatanib	[++]		z	
	Ethacrynic acid	[++]			1
SCN1A	Bisacodyl	[++]	x		
SLC2A1	Buspirone	++	x		
	Metformin	[++]		z	
	Deferoxamine	[++]	xx		
	Fluphenazine	[++]	x		
SMAD3	Isotretinoin	**++**	xx		1
	Fluphenazine	**++**	xxx		
SPAST	Naltrexone	[++]	xx		
	Fluphenazine	**++**	xx		
FBN2	Dexamethasone	**++**		z	
LIMS1	Isotretinoin	[++]	x		
SLC39A14	Bisacodyl	[++]	x		
	Acetylcysteine	+		z	
AGPAT2	Mexiletine	+	x		n/a
AGXT	Theophylline	**++**	x		n/a
	Trazodone	**++**	x		n/a
AMACR	Fluphenazine	+	xx		n/a
ASAH1	Fluphenazine	**++**	xx		
ATP7A	Bisacodyl	[++]	x		
BCKDHB	Chlorpropamide	+	x		
CLN3	Bisacodyl	[++]	x		
CTSA	Bisacodyl	[++]	x		
GALC	Bisacodyl	[++]	x		
	Mexiletine	+	x		
GALNS	Bisacodyl	[++]	x		
GUSB	Idarubicin	+			1
HEXA	Idarubicin	+			1
	Bisacodyl	[++]	x		
HEXB	Bisacodyl	[++]	x		
HPRT1	Chlorpropamide	+	x		
HSD11B2	Calcitriol	+		z	n/a
IDS	Bisacodyl	[++]	x		
	Naltrexone	[++]	x		
	Fluphenazine	**++**	x		
IDUA	Buspirone	+	x		n/a
MAN2B1	Idarubicin	+			1
	Dacarbazine	**++**	x		
NEU1	Bisacodyl	[++]	x		
	Fluphenazine	**++**	xxx		
SGSH	Mexiletine	+	x		n/a
TYMP	Calcitriol	[++]		z	
CSF1R	Dexamethasone	+		z	n/a

++ = robust response (>75% induction),+ = modest induction (40–74%)

[++] = induction only observed with high dose of drug (therapeutic dose)

x = response observed in one cell line, xx = two cell lines, xxx = three cell lines

z = identified by causal reasoning engine algorithm

1 = identified by fibroblast screen

n/a = not applicable as screen was not performed on this gene

The remaining blank cells indicate that a gene-drug interaction was not demonstrated/predicted based on CMAP, CREKB or FScreen.

### Causal reasoning engine knowledge base mining

The medical literature mining algorithm to identify drug-gene relationships was initially set at Edge 2 low stringency settings (see material and methods) but generated so many leads that there was little value in terms of identifying true interactions (data not shown). At the more stringent level focused at exclusively “Edge 1” effects of the 23,250 potential drug-gene interactions (75 genes × 310 drugs), 119 different putative interactions were identified (0.6%; Supplementary Table 2; column LF).

### Cell based screening

310 drugs were tested for their impact on 51 target genes in normal human fibroblasts as outlined in methods. Based on the stringent statistical criteria used for identifying putative positive drug-gene interactions (*Z*-score > 1.65 for both pools of five containing a given drug), of 15,810 interactions tested in our cell-based screen, 61 putative interactions were identified (0.4%; Supplementary Table 2, column LG).

### Validation of hits

**Fig. 1:**
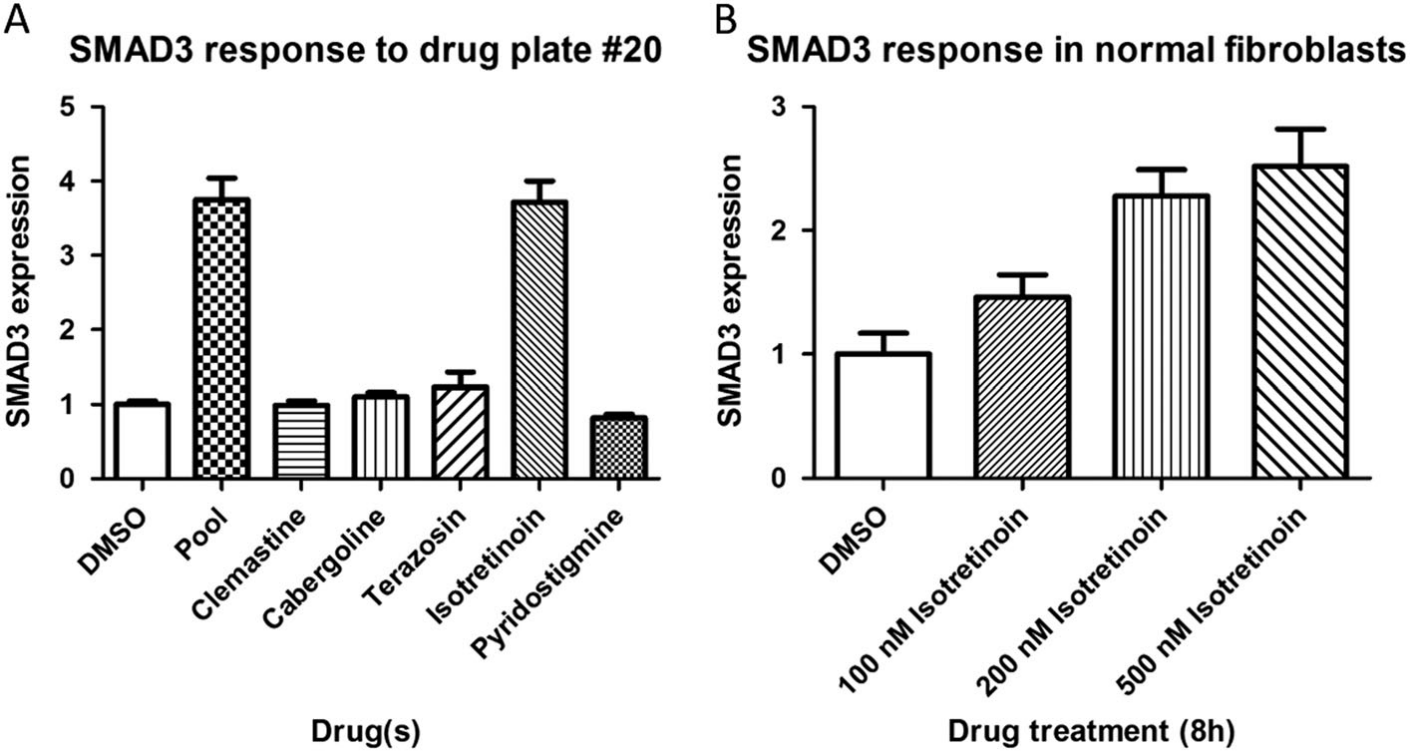
**a** Deconvolution of drug pool associated with a SMAD3 induction in fibroblasts. qPCR data displaying relative expression of SMAD3 in response to drug pool 20 and its individual component drugs. All drugs are at 2 uM concentration in dimethyl sulfoxide. The pool response was successfully validated and single drug analysis revealed that this pool response was due to the drug Isotretinoin. **b** A dose response SMAD3 mRNA Isotretinoin in fibroblasts

**Fig. 2:**
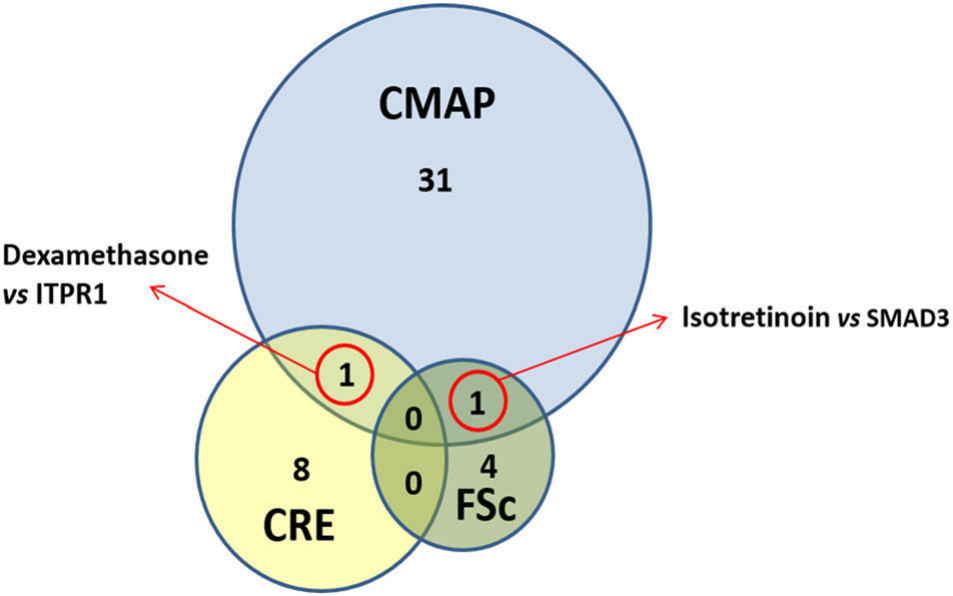
Venn diagram showing the overlap of the validated drug-gene interactions identified by the three methods. Thirty four of 685 CMAP hits tested, 9 of 85 CREKB hits and 5 of 55 hits identified in the fibroblast cell-based screen validated by qRT-PCR measurement of target mRNA in single drug dosing (2 uM) of fibroblasts for 8 h. (Table 2)

**Fig. 3:**
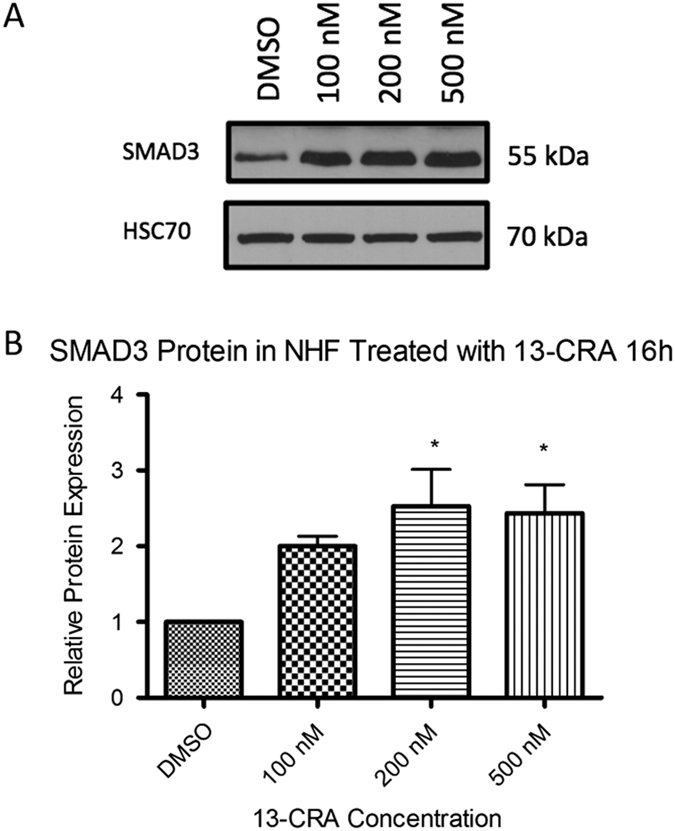
**a** Sample western blot for SMAD3 protein in NHF treated with various concentrations of isotretinoin (13-*cis*-retinoic acid; 13-CRA) for 16 h. **b** Quantified SMAD3 protein expression. Error bars represent SEM. *n* = 4. Heat shock cognate protein 70 (HSC70) *p < 0.05

Validation of a subset of the hits identified by one or more of the three methods (685/970 for CMAP, 85/119 for CRE; 55/61 for the cell-based screen) was undertaken in human skin fibroblasts by incubating with single drug (2  uM and drug doses closer to therapeutic levels) for 8 h followed by qRT-PCR measurement of target mRNA. In total, of the 685 CMAP hits tested, 34 validated in fibroblasts (5%), for the 85 CRE hits tested, 9 validated (11%) and of the 56 fibroblast screen hits tested, 5 validated (9%). Overall, 47 different drug-gene interactions validated (Table 2), and in only two cases; isotretinoin vs. SMAD3 (CMAP and fibroblast screen; Fig. 1) and dexamethasone vs. ITPR1 (CMAP and CRE) was the same interaction identified by more than one method (Fig. 2). Of these 47, 13 demonstrated a robust response with therapeutic levels (serum concentration) of the drug, another 13 showed a modest response, and 21 displayed a response only when the drug dose was very high (typically 10-fold or greater than therapeutic levels). Furthermore, 19 drugs accounted for these 47 responses (see Table 2). Although transcriptionally active compounds including the vitamin A analog isotretinoin (2 genes), dexamethasone (3 genes), calcitriol (3 genes) were among the agents which upregulated mRNAs, some on the transcriptomic modulating list were unexpected; suprapharmacologic levels of the laxative bisacodyl induced a full 11 target mRNAs while the antipsychotic phenothiazine, fluphenazine upregulated 7. Given one of the most robust responses was observed with isotretinoin and SMAD3, we next attempted to see if there was a corresponding protein induction; a greater than 2-fold induction was seen with 100–500 nM isotretinoin (Fig. 3).

## Discussion

The era of next generation DNA sequencing based rare disease gene identification has served to underline the comparative dearth of effective rare disease therapies; fewer than 500 for the thousands of disorders; the expense of many is such that payers around the world are struggling and the potential to fund many more is clearly in doubt. New means of identifying inexpensive rare genetic disease therapies are clearly needed. As one possible solution, rare diseases are approached here as problems of gene dosage; conditions resulting from too little or too much of a given protein activity. We set out to establish the frequency with which drug-gene interactions (in this case, pharmacologic upregulation of mRNA) can be first identified and then validated as a possible therapeutic approach to attenuate this pathogenic dysregulation for a targeted group of rare diseases. We searched an extensive transcriptomal database (Connectivity Map (CMAP); 149 drugs; 70 genes), used a text mining platform (causal reasoning engine; 310 drugs; 75 genes) and conducted a targeted drug screen on normal human fibroblasts (310 drugs; 51 genes). Forty-seven examples of drug induction of potential rare disease modifying genes were identified in this fashion.

Although we assessed 75 genes/76 disorders, it is clear that there exist many others that could be assessed, in particular the so called hypomorphs. In theory any recessive disorder with missense mutations that preserve both the correct protein localization and some function could be a potential target. Broadly speaking 28% of 7000 genetic disorders are recessive (http://ec.europa.eu/health/rare_diseases/orphanet/report_en) and approximately 70 percent are missense.^17^ It is not known what fraction of these preserves some function and correct cellular targeting but even it were as low as 25% there are over three hundred such disorders and this may be an underestimate.

In terms of the initial identification of potentially significant drug-gene interactions, the CMAP had, by far, the greatest proportional yield of the three approaches; 9.3% (970/10,430) were potentially positive compared with only 0.6% (119/23,250) and approximately 0.4% (61/15,810) for the CREKB mining and cell-based assay, respectively. It should be noted that although the CMAP appeared much more effective than the other two approaches in identifying putative positives, an interaction was scored as a positive if just one cell line (of the three tested) showed a response; if only those that showed a response in all three lines were counted, the yield dropped to 1%, much closer to the values obtained for the other two platforms.

The proportion of the putative positive drug-gene interactions which were next validated (qRT-PCR amplification of the specific mRNA from normal human fibroblasts grown in 2 uM drug) was more consistent; approximately 5% of cases for the CMAP, 11% of the CREKB drug-gene interactions and 9% for our targeted drug screen validated (Table 2; Supplementary Table 2; columns LC-LK). These relatively low validation rates likely devolve from a number of factors; in the case of CMAP, the cells were transformed and not primary lineages and the drug concentration was comparatively high (10 uM). The CREKB showed the highest validation rate at 11%, closely followed by the targeted drug screen (9%). Nonetheless approximately 90% of putative hits did not validate. In the case of CREKB, the inference of a direct drug-gene interaction is based on medical literature encompassing a wide range of tissue and cell types with different drug concentrations and timing; thus the one in ten validation rate might be expected. With regard to the fibroblast screen, the 9% validation rate was unanticipated; it may be that the combination of drugs in pools of 5 to achieve the greatest throughput resulted in unpredictable synergistic multi-drug effects that increased the false positive rate. Conversely, it is also likely that repression of gene expression by individual drugs may have masked drug pool induction events resulting in false negatives.

Nevertheless, 47 different drug-gene interactions were identified by the three different methods, showing that these approaches can be used in a complementary manner. The methodologies themselves have significant differences; CREKB is mined from a medical literature often directed toward specific diseases, drug classes and specific readouts while the system wide gene expression data found in CMAP are from cells that are transformed with all the genetic anomalies that that may entail. Interestingly if one looked exclusively at the CMAP drug-gene interactions in which all three cell lines were positive, 18% (2/9) validated; the highest yield by a significant margin. Finally, the normal human skin fibroblasts used in the FDA screen while a technically tractable cell line express only approximately two thirds of the transcriptome over 80% of which encode proteins expressed in all tissues (i.e., “housekeeping proteins”).^18^

The mining of existing computational datasets for drug repurposing has been explored by other groups. In particular, a number of reports using the CMAP have been published, focusing on more common conditions such as dyslipidemia,^19^ pain,^20^ cancer,^21^ inflammatory bowel disease,^22^ cachexia,^23^ osteoporosis,^24^ and alopecia.^25^ A recently published systematic assessment of the CMAP mining for drug repurposing for cancer and other common diseases showed predictive utility particularly for cancer.^26^ Our study is the second of which we are aware that uses the CMAP to study rare genetic diseases ^27^ and unique in that it utilizes single gene levels rather than gene signatures as the therapeutic target. Similarly the causal reasoning engine platform ^12^ has been used by a number of groups to look for established drugs with activity against cancer and pain;^28^ it has not to our knowledge been used to look for gene induction or applied to rare diseases.

Having identified 47 possible leads, we shall next assess for a protein upregulation reflecting mRNA induction; we have shown this for a number of conditions (e.g., SLC2A1 by deferasirox, SMAD3 by isotretinoin; Fig. 3) and would anticipate that, despite a considerable attrition, a significant proportion of the remainder shall as well.^29, 30^ Following this assessment in vivo analysis for induction in the tissue of greatest pathophysiologic relevance will be undertaken.

Finally, although the concept of increasing an underexpressed protein to treat rare genetic disease is intuitively attractive, insight into the biology of the disease process will also be critical for the next stage of assessment. For example Loeys Dietz type III aortopathy despite being caused by haploinsufficiency of the TGFβ responsive SMAD3 transcription factor,^31^ counterintuitively results in an induction of the TGFβ axis.^32^ Treatment of young SMAD3 ± mice with SMAD3 inducing retinoic acid thus appears only to add to TGFβ activation and not to improve but possibly exacerbate their aneurysmal phenotype. The timing of the repletion (earlier rather than later) appears critical to the possibility of benefit (E. MacFarlane; personal communication).

In another example, autosomal-recessive intellectual disability with cerebellar atrophy syndrome (also known as congenital disorder of glycosylation, type II) is caused by a comparative manganese deficiency resulting from mutation of the manganese transporter gene SLC39A8.^33^ We anticipated induction of the paralogous manganese transporting SLC39A14 would be beneficial at the outset of this study, but recent work has shown that loss of this transporter results in manganese accumulation.^34^ The gene is an exporter of manganese via hepatic metabolism and its upregulation might actually only worsen the manganese depletion observed in the syndrome; downregulation of the SLC39A14 may therefore be the desired outcome. It is clear that after a promising drug gene induction is observed, validation in the appropriate animal disease model is critical.

In conclusion, two in silico approaches and a directed drug screen configured to identify putative drug-gene (mRNA) interactions for rare genetic diseases has provided new putative target drugs that may be further tested for in vivo protein induction as a prelude to potential use as repurposed rare disease therapeutics. Our experience with pharmacologic modulation of gene activity will help frame expectations for those pursuing this path and the data presented here will serve as a useful resource for those studying specific diseases and genes.

## Methods

### Drug selection

Although the Connectivity Map employed 1300 compounds ^9, 10^ and the CREKB subtends ~ 450,000 causal relations (12), for purposes of tractability the definition of a subset of compounds was needed. Given that repurposed medications for rare disorders will likely have to be given for life, 310 drugs were therefore selected for testing from the Screen-well v2 FDA approved drug library (Enzo Life Sciences) using overall safety profile and tolerability as desirable characteristics (Supplementary Fig. 1). The impact of these 310 drugs on 75 genes as elicited by two and, where possible, all three approaches therefore comprised the central focus of our work.

### Mining of existing CMAP datasets

Broad Institute’s CMAP (https://www.broadinstitute.org/cmap/) is a database of drug-gene interactions employing the Affymetrix GeneChip U133-A microarray-based transcriptomic analysis of mRNA extracted from three cell lines (HeLa, PC3, and MCF7) individually treated with ~ 1300 drugs for 6 h at a concentration of 10 µM.^9, 10^ All drugs demonstrating mRNA induction for each of the genes represented in this dataset were identified (70/75 target genes; Table 1). These were then cross-referenced with our FDA drug panel identifying 149 drugs that were utilized in the CMAP screen.

### Mining the CREKB

Given the extensive and increasingly detailed biomedical literature, text-mining has become a promising approach with which to identify putative drug-gene interactions.^35^ The Pfizer CREKB integrates microarray transcriptomic data with causal statements derived from the biomedical literature (Ingenuity and Selventa “knowledge bases”) to infer upstream molecular mechanisms mediating observed gene expression changes.^11, 12^ The underlying knowledge base (CREKB) was used to predict pathway targets that would impact the expression of the disease-related mRNA for our target 75 disease genes for the 310 drugs under test. The interactions were then classified by the proximity of their relationship. Edge 1 effects refer to a direct link, whereas edge 2 is inferred from indirect relationships (e.g., when considering drug A and gene C, a direct impact of A on C is an edge 1 effect while drug A affecting gene B which regulates gene C is an edge 2 effect.) However, edge 2 effects were extremely common therefore only edge 1 direct links were scored. This approach was able to distinguish directionality in terms of the relationship, i.e., up-regulation of gene targets by drugs vs. repression.

### Fibroblast-based drug screening

Pooled drugs (5 per well) at 2 uM each were utilized; drugs were assessed twice in the screen, and never pooled with the same drugs (Supplementary Table 1). Dosing was conducted on normal human fibroblasts at an early passage (10) grown to 100% confluence in 10 cm dishes to minimize differential responses devolving from the cycling state of the cell. Cells were collected after an 8 h treatment via trypsin and RNA isolated by RNeasy MICRO with on-column DNAsing (QIAGEN). Minimal toxicity was observed by light microscopy at 8 h. cDNA was synthesized via the iScript Advanced system (BIO-RAD) then transferred to a 384-well plate and qPCR performed using the iQ Supermix (BIO-RAD) on a CFX-384 instrument (BIO-RAD). Each sample was represented in triplicate wells (technical replicates). In total, qPCR was performed on 51 different target genes of interest (Supplementary Table 2; column B). The remaining genes were not tested due to very low/negligible expression in fibroblasts. In addition GAPDH and YWHAZ were also run to determine relative stability of signal and cDNA concentration. Relatively low signals (Ct values > 27) were observed for 20/53 genes analyzed.

The drug screen was configured so that a single 384-well qPCR plate contained all 128 drug pools (5 drugs per pool) comprising our library in triplicate; thus a single gene was measured in each of the 53 qPCR plates (51 genes tested). Each 384 well plate thus had the same array of drug pools; it was noted that some pools had tendency to either consistently up or downregulate irrespective of the gene being tested. Although the source of the “hot” and “cold” drug pools is unclear, a plate correction was conducted; an individual drug pool-gene reading was normalized against the geometric mean of all genes tested for the corresponding drug pool in all other plates (i.e., 53 qPCR Ct values per drug pool). *Z*-scores were then calculated for each gene dataset. Each drug of the 310 drug panel was represented twice in the pools of five; an mRNA level demonstrating a *Z*-score greater than 1.65 (*p* value < 0.05) for a given drug in both pools was identified as a putative hit warranting additional investigation and validation.

### Western blot validation of isotretinoin

Following treatment of fibroblasts (NHF) with isotretinoin, cells were lysed using radioimmunoprecipitation assay buffer supplemented with protease and phosphatase inhibitors by sonicating for 30 s followed by a resting period of 30 s, repeated for a total of 8 min using a water bath sonicator (DiaMed Transsonic T460). Samples were then centrifuged at 4 °C for 40 min and protein was quantified using a Bradford Protein Assay (Bio-Rad #500-0006). 40 μg of protein was loaded on 11% acrylamide gels and run at 80 V for 30 min, then 120 V for 1 h before being transferred with a Bio-Rad Semi-Dry Transfer system (Amersham Biosciences TE 77 Semi-Dry Transfer Unit) onto nitrocellulose membranes (Bio-Rad, #162-0115) for 1 h 15 min at 65 milliAmps/gel. Membranes were blocked in PBS/Tween (0.05% Tween-20) with 5% dried skimmed milk for 1 h. Anti-SMAD3 antibody (Abcam #ab40854) was used at 1:3000 in PBS/Tween (0.05% Tween-20) with 5% dried skimmed milk powder overnight at 4 °C. Anti-rabbit secondary antibody was used at 1:5000 (Cell Signaling Technology, #7074S) in PBS/Tween (0.05% Tween-20) with 5% dried skimmed milk powder at room temperature for 1 h. Both primary and secondary antibody washes were followed by three 15-minute washes with PBS/Tween (0.05% Tween-20). Antigen detection was carried out using Clarity (Bio-Rad, #170-5061) according to the manufacturer’s instructions. Loading control anti-HSC70 (Santa Cruz Biotechnology, #sc-7298) was used at a concentration of 1:2000 in PBS/Tween (0.05% Tween-20) with 5% dried skimmed milk powder at room temperature for 1 h. Anti-mouse secondary antibody was used at 1:5000 (Cell Signaling Technology, #7076S) in PBS/Tween (0.05% Tween-20) with 5% dried skimmed milk powder at room temperature for 1 h. Blots were quantified using ImageJ 1.48 v software.

### Availability of data, materials and methods

The CMAP database is available at: https://www.broadinstitute.org/cmap/ The Pfizer CREKB was made available to us through collaboration.

## Electronic supplementary material

Supplementary Table 1

Supplementary Table 2
